# Biocompatibility and osteogenic properties of porous tantalum

**DOI:** 10.3892/etm.2015.2208

**Published:** 2015-01-23

**Authors:** QIAN WANG, HUI ZHANG, QIJIA LI, LEI YE, HONGQUAN GAN, YINGJIE LIU, HUI WANG, ZHIQIANG WANG

**Affiliations:** 1 Graduate School, Southern Medical University, Guangzhou, Guangdong 510515, P.R. China; 2 Department of Anatomy, Basic Medical College, Hebei United University, Tangshan, Hebei 063000, P.R. China; 3 Department of Joint Surgery, The Second Hospital of Tangshan, Tangshan, Hebei 063000, P.R. China; 4 Experimental Center, Hebei United University, Tangshan, Hebei 063000, P.R. China; 5 Chongqing Runze Pharmaceutical Co. Ltd., Chongqing 401120, P.R. China; 6 Department of Orthopedics, Affiliated Hospital of Hebei United University, Tangshan, Hebei 063000, P.R. China; 7 Department of Hand Surgery, The Second Hospital of Tangshan, Tangshan, Hebei 063000, P.R. China

**Keywords:** porous tantalum, osteogenesis, biocompatibility, cell toxicity, bone tissue engineering

## Abstract

Porous tantalum has been reported to be a promising material for use in bone tissue engineering. In the present study, the biocompatibility and osteogenic properties of porous tantalum were studied *in vitro* and *in vivo*. The morphology of porous tantalum was observed using scanning electron microscopy (SEM). Osteoblasts were cultured with porous tantalum, and cell morphology, adhesion and proliferation were investigated using optical microscopy and SEM. In addition, porous tantalum rods were implanted in rabbits, and osteogenesis was observed using laser scanning confocal microscopy and hard tissue slice examination. The osteoblasts were observed to proliferate over time and adhere to the tantalum surface and pore walls, exhibiting a variety of shapes and intercellular connections. The porous tantalum rod connected tightly with the host bone. At weeks 2 and 4 following implantation, new bone and small blood vessels were observed at the tantalum-host bone interface and pores. At week 10 after the porous tantalum implantation, new bone tissue was observed at the tantalum-host bone interface and pores. By week 12, the tantalum-host bone interface and pores were covered with new bone tissue and the bone trabeculae had matured and connected directly with the materials. Therefore, the results of the present study indicate that porous tantalum is non-toxic, biocompatible and a promising material for use in bone tissue engineering applications.

## Introduction

Autograft and allograft transplantations are the primary treatments for large bone defects caused by trauma, tumor resection and infection; however, the problems of limited bone sources and immune rejection or infection persist. By contrast, metallic biomedical materials are playing an increasingly important role in the treatment of bone defects. However, since these metals and their alloys do not exhibit the same elastic modulus as bone, stress shielding occurs, resulting in relative displacement of metal implants and friction along the metal-bone interface (1,2). This friction generates debris, inducing a macrophage response, which causes deterioration or absorption of the bone tissue and ultimately leads to loosening of the implant or fracture of the host bone (3–5). Therefore, further research is required to enhance the *in vivo* longevity of metallic biomedical materials.

To date, research in this area has focused primarily on porous titanium and its alloys. However, the clinical application of porous metal materials has remained limited due to their low porosity, small surface friction coefficient and concerns regarding their elastic modulus (6–8). Porous tantalum has been reported as a promising material for use in bone tissue engineering. However, only Zimmer Corporation (Warsaw, IN, USA) has produced porous tantalum material with high porosity (75–80%), good tissue compatibility, a large surface friction coefficient and the proper elastic modulus that closely matches that of human bone. As a result, porous tantalum has been widely applied in arthroplasty, spinal fusion surgery and the treatment of femoral head necrosis (9–12). However, the widespread use of porous tantalum in China is limited by the high price. Cooperating with numerous domestic research institutions, Chongqing Runze Pharmaceutical Co., Ltd. (Chongqing, China) have successfully developed porous tantalum materials, which were prepared by slip-casting powder through teeming technology. The porous tantalum selected was −250 mesh pure tantalum powder with a certain amount of additives and added sponge carrier to control the pore diameter of porous materials, porosity and pore distribution, and approved by slip-casting forming and through 1,500–2,100°C high temperature sintered and post-treatment technology of necessary preparation (preparation method has been submitted for patenting). The biomechanical indexes showed that Chinese porous tantalum possessed high porosity, an appropriate pore diameter size and exhibited biomechanical performance characteristics that were comparable to human bone, suggesting that tantalum may be an ideal bone graft material (Table I).

The present study investigated the physical properties and cytotoxicity of Chinese porous tantalum and examined osteogenesis at the tantalum-host bone interface following implantation in the lateral epicondyle of a rabbit femora. The aim of the study was to assess the possibility of the wider application of Chinese porous tantalum material.

## Materials and methods

### Materials

Eight fetal New Zealand rabbits and 24 adult male rabbits (weight, 2.5–3 kg) were provided by the Experimental Animal Center of Hebei United University (Tangshan, China). Porous tantalum was obtained from Chongqing Runze Pharmaceutical Co., Ltd. The porous tantalum selected was −250 mesh pure tantalum powder with a certain amount of additives and added sponge carrier to control the pore diameter of porous materials, porosity and pore distribution, and approved by slip-casting forming and through 1,500–2,100°C high temperature sintered and post-treatment technology of necessary preparation (preparation method has been submitted for patenting). Low-glucose Dulbecco’s modified Eagle’s medium (DMEM) was purchased from Gibco Life Technologies (Carlsbad, CA, USA), 3-(4,5-dimethylthiazol-2-yl)-2,5-diphenyltetrazolium bromide (MTT) reagent was purchased from Sigma-Aldrich (St. Louis, MO, USA) and an inverted phase contrast microscope was obtained from Nikon Corporation (Tokyo, Japan). In addition, a scanning electron microscope (JSM-6030LV; JEOL, Tokyo, Japan), a Leica SP1600 hard tissue slicer (Leica Microsystems GmbH, Wetzlar, Germany), a laser scanning confocal microscope (Olympus Corporation, Tokyo, Japan) and a Bio-Rad 680 microplate reader (Bio-Rad Laboratories, Hercules, CA, USA) were employed. This study was approved by the Institutional Animal Care and Use Committee of Hebei United University. Postoperative care was professionally managed by trained personnel and supervised by veterinarians.

### Osteoblast cultivation

The periosteum and soft tissue surrounding the crania of the fetal New Zealand rabbits were removed and cut into 1xl mm pieces prior to digestion in a solution of 0.25% trypsin and 0.1% type II collagenase. Following centrifugation at 2,012 × g for 10 min at 37°C, the supernatant was discarded and DMEM containing 10% fetal bovine serum (Gibco Life Technologies) was added to the white precipitate, which was pipetted into a cell suspension for counting the total number of cells and then seeded into cell culture dishes. The cells were cultured with DMEM supplemented with 10% fetal bovine serum in a humidified incubator containing 5% CO_2_ at 37°C. Primary osteoblasts were passaged after reaching 80–90% confluence. Osteoblasts were identified by alkaline phosphatase (ALP) staining, collagen type I immunocytochemical staining and calcium nodule staining.

### Cytotoxicity evaluation

Porous tantalum extract was prepared according to ISO 10993-5, 2009 for high pressure disinfection of porous tantalum extract preparation (13). Extracts were prepared based on the principle of ‘material weight/extraction transmitter = 0.2 g/ml’, which represented 100% concentration of the extract. Extraction conditions were 37°C in 5% CO_2_ for 72 h, and metal ions were precipitated. Once the extraction was completed, sterilization was achieved by passing the extract through a 0.22-μm filter.

An MTT assay was conducted to determine the effects of porous tantalum on osteoblast viability, growth and proliferation (14). Third passage osteoblasts were suspended and seeded in a 96-well plate at 2×10^4^ cells per well. Porous tantalum extract (experimental group) or complete medium (control group) was added to the wells until the volume in each well was 200 μl. Next, 20 μl MTT (5 mg/ml) solution was added to each well (pH 7.4) and the plate was incubated for 4 h in the CO_2_ incubator. Dimethyl sulfoxide was added and low-speed oscillation was applied. A Bio-Rad enzyme-linked immunometric meter (measurement wavelength, 490 nm; reference wavelength, 650 nm) was used to detect the optical density (OD) value of the solution in each well, from which a cell growth curve was prepared.

### Osteoblast culture on porous tantalum to evaluate cytocompatibility

Pancreatin was used to digest a proportion of the osteoblasts in order to adjust the cell concentration to 1×10^6^ cells/ml. Subsequently, a 30-μl cell suspension was seeded onto each presterilized porous tantalum sample in the culture plates. The porous tantalum was turned over and the cells were seeded onto the other side. DMEM containing 10% fetal bovine serum was added, and samples were cultured at 37°C in 5% CO_2_ and saturated humidity. Cell growth and adhesion to the tantalum samples was observed on an inverted phase contrast microscope every day.

Sterilized tantalum samples and those on which cells were seeded were collected on days 3, 5, 7 and 10. The samples were washed with phosphate-buffered saline, fixed in 2.5% glutaraldehyde, dehydrated in 30–100% t-butyl alcohol solutions, dried in a 37°C electric thermostatic drier (JEOL JFC-1600) for 24 h and underwent ion sputter metal spraying for scanning electron microscopy (SEM) observation of cell adherence and growth on the material.

### Preparation of rabbits for porous tantalum implantation

**Figure 1:**
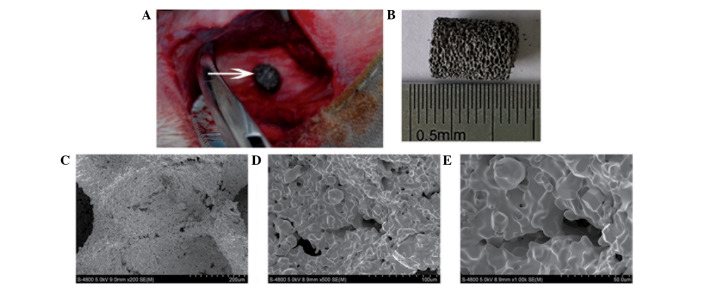
Model and physical properties of porous tantalum. (A) Intraoperative photograph of the bone defect experiment showing the porous tantalum implantation model in the rabbit femoral condyles. The bone defect was filled with porous tantalum (arrow). (B) Appearance of porous tantalum. (C-E) Pore morphology of the porous tantalum specimen, with a rough and irregular surface and interconnected pores with diameters of 400–600 μm. (C) Material surface and cross-section show interconnected pore distribution, with a pore diameter size of 200–400 μm (scale bar, 200 μm). (D) Trabecular pillar exhibits a micropore structure, with the diameter of the interval of the trabecular pillar ~100 μm (scale bar, 100 μm). (E) Micropore structure of the trabecular pillar, where the diameter of the micropores was ~10 μm. (Scale bar, 50 μm).

A total of 24 male adult New Zealand white rabbits (weight, 2.5–3.0 kg) were selected. The model of porous tantalum implantation in the femoral condyle was established using improved surgical methods, as reported in a previous study (15). Briefly, 20% urethane (3–4 ml/kg) was injected into the ear vein for anesthesia. The vastus lateralis muscle was cut from the outside part of the lower femur, along the femoral shaft, to expose the femoral condyle. A 3-mm Kirschner skeletal wire [Association for Osteosynthesis(AO)/Association for the Study of Internal Fixation (ASIF), Davos, Switzerland]was used to drill into the femoral lateral condyle, which was ~1 cm from the knee, perpendicular to the femoral shaft. The drilling resulted in a cylindrical bone tunnel with a diameter of ~3 mm and a depth of 5 mm. A porous tantalum rod (diameter, 3 mm; height, 5 mm) was implanted in the bone along the tunnel, with the tantalum rod contacting as closely as possible with the tunnel wall of the host bone (Fig. 1A). The wound was sutured and marked with adequate hemostasis. Three days after surgery, the wound was disinfected and penicillin was injected intramuscularly. Four animals were injected with luciferin calcein solution (10 mg/kg) in the gluteus maximus on day 5 after surgery. Fluorescein alizarin red solution (40 mg/kg) was injected in the same manner on day 19 after surgery, in order to observe osteogenesis. The animals were separated into cages, without limitation of movement, and euthanized at week ten after surgery.

### Porous tantalum-bone hard tissue slicing and staining

In total, 20 rabbits were euthanized by air embolism at weeks 2, 4, 8 and 12 after surgery (five at each time point). The bone tissue in the femoral condyle was removed along with the porous tantalum rod, and then washed, trimmed and fixed in 10% formaldehyde. Following dehydration, infiltration, embedding (20 g benzoyl peroxide in 800 ml methacrylic acid methyl ester, phthalic acid and 200 ml dibutyl) and polymerization, a metal slicer was used to slice the material along the direction parallel to the longitudinal axis of the porous tantalum rod, fully exposing its plane. The plane was polished and 90-μm slices were prepared, which were ground down to 20 μm, dried and exposed to glycol ether ester to remove any plastic. Sections were stained with methylene blue and osteogenesis was observed under a light microscope. The materials acquired at week 10 after the surgery were processed into porous tantalum-bone hard tissue slices using the same method and were used to evaluate osteogenesis at the porous tantalum-bone interface via laser scanning confocal microscopy with a 488-nm laser (calcein excitation wavelength) and a 543 nm laser (alizarin red excitation wavelength).

### Statistical analysis

Data were analyzed using SPSS 20.0 statistical software (IBM, Armonk, NY, USA). Experimental data are expressed as the mean ± standard deviation. In the cytotoxicity and cell growth experiments, comparisons between the experimental and control groups were analyzed using the Student’s t-test. P<0.05 was considered to indicate a statistically significant difference.

## Results

### Surface characteristics of porous tantalum

Under microscopic observation, the appearance of the porous tantalum scaffolds was gray and smooth, with pinpoint-sized pores distributed evenly in a honeycomb pattern on the surface and fracture surface (Fig. 1B).

SEM revealed that the material surface and cross-section had an interconnected pore distribution, with a pore diameter size of 400–600 μm. The trabecular pillars had a microporous structure, where the diameter of the micropores was ~100 μm. Pore distribution was uniform and the pores were interconnected. The microporous structure with interconnected pores had a diameter of 200–400 μm, the particle diameter was 20–50 μm and there were 50–200 μm interstices between the particles (Fig. 1C–E).

### Cultivation of isolated fetal rabbit osteoblasts

Primary osteoblasts were observed as translucent spheres under an inverted phase contrast microscope. The cells grew adherently after 24 h, presenting a variety of forms, which were primarily short fusiform and triangular with the cytoplasm extending outward to form protrusions. The cell volume was enlarged by day 3, with cells connecting and increasing in number. By day 7, the cell groups fused to form a monolayer covering the bottom of the plate, and the cell morphology became polygonal or irregular. The cells were passaged when they reached 80–90% confluence. The majority of the cells exhibited triangular or long fusiform shapes, with the cell volumes and growth rate increasing. The third passage cells exhibited a long fusiform morphology. The results of ALP staining, collagen type I immunocytochemical staining and calcium nodule staining were positive, confirming that the cells were osteoblasts.

### Effects of porous tantalum extract on osteoblast viability and growth

**Figure 2:**
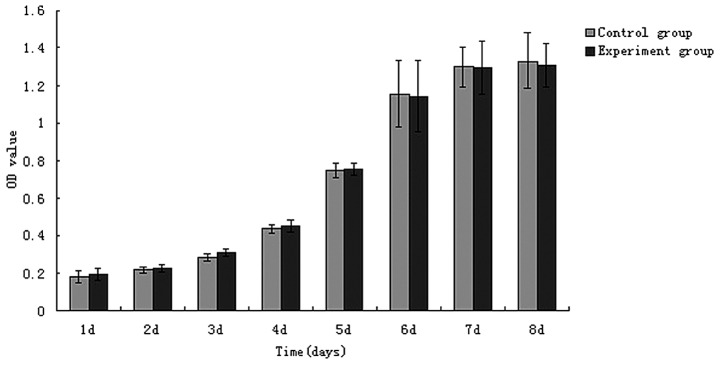
3-(4,5-dimethylthiazol-2-yl)-2,5-diphenyltetrazolium bromide assay results demonstrating the effect of tantalum extract on osteoblast proliferation. Osteoblast cell proliferation efficiency of the groups treated with (experimental) or without (control) porous tantalum extract between days 1 and 8 of cell culture. Proliferation of experimental group cells changed from slow to rapid, and then to slow again, ultimately entering the stable phase, between days 1 and 8 of osteoblast culture. Cell proliferation did not statistically differ between the groups with extended cultivation time. Independent sample t-tests: P>0.05, vs. control group. OD, optical density.

Absorbance values in the MTT assay showed that between days 1 and 8 of osteoblast culture, osteoblast growth changed from slow to rapid, and then to slow again, ultimately entering a stable phase. Absorbance values for the control cells and those exposed to porous tantalum extract at the same time points were analyzed using an independent sample t-test, and the results showed that the cell proliferation did not statistically differ between the groups with extended cultivation time (P>0.05). A cell growth curve was constructed based on the OD values (Fig. 2). The results demonstrated that tantalum metal ions were not cytotoxic to osteoblasts, achieving the basic requirement of implant materials.

### Osteoblast culture on porous tantalum samples for evaluation of cytocompatibility

Observation by inverted phase contrast microscopy showed that in osteoblasts cultured on porous tantalum for one day, a small number of cells began to adhere to the edge of the material. In addition, cells of different sizes were dispersed on the bottom of the culture plates, with cell processes exhibiting fusiform or polygonal shapes. The number of cells adhering to the edge increased and the cells proliferated rapidly between days 3 and 5, aggregating and fusing into flakes. Monolayers of cells were formed in the bottom of the wells after seven days.

**Figure 3:**
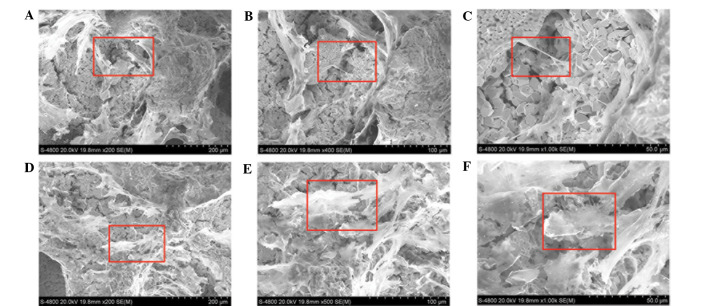
Scanning electron microscopy images of osteoblasts cultured on tantalum. (A-C) By day 5, adjacent cells connected with each other across the pores, creating a flake with burr-like projections extending out to the surroundings (scale bars for A-C are 200, 100 and 50 μm, respectively). (D-F) By day 10, cells on the surface and in the pores grew into multiple layers, secreting matrix and covering the surface completely (scale bars for D-F are 200, 100 and 50 μm, respectively).

SEM revealed that the porous tantalum had numerous pores. A small number of cells with varying morphologies were sparsely arranged on the tantalum samples. By day 3, a few protrusions grew adherently on the surface of the scaffolds and within the scaffold pores. The cells stretched, lengthened and extended pseudopodia gradually over time. By day 5, the processes of adjacent cells connected with each other across the pores, creating a flake with burr-like processes extending into the surroundings. On day 10, cells on the surface and in the pores grew into multiple layers, secreting matrix and covering the surface completely (Fig. 3).

### Morphological observations of the porous tantalum implanted in the rabbit femoral condyle

Animals were conscious 30 min after the surgery and exhibited recovery of knee movement. The incisions exhibited no swelling, bleeding or oozing, and the wounds healed well. Loosening of implantation materials was not observed. Implanted porous tantalum rods were closely combined with the host bone at each time point. No gap between the host bone and the tantalum implant was visible by the naked eye and no adverse reactions, such as infection, occurred.

Observation of osteogenesis at the tantalum-host bone interface in the hard tissue slices revealed that the porous tantalum was in close contact with the bone tissue, as shown by methylene blue staining.

**Figure 4:**
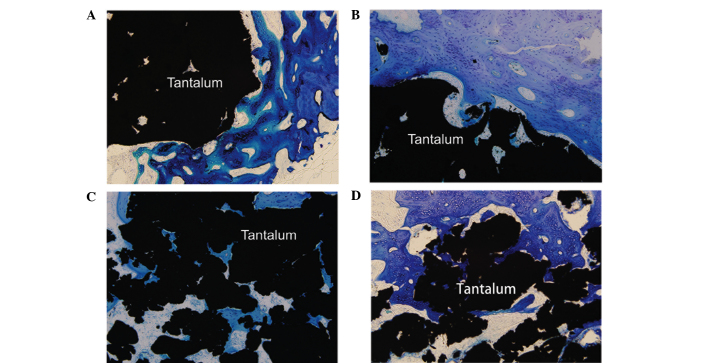
Histological observation of hard tissue slices prepared from porous tantalum implants at weeks (A) 2, (B) 4, (C) 8 and (D) 12 after implantation (methylene blue staining; magnification, ×100).

During week 2, new bone tissue grew at the interface of the tantalum and the host bone, on the surface of the material and in the pores, with small blood vessels also growing in certain areas. By week 4, the number of new bone cells at the interface increased, and the cells connected with the host bone as flakes. Bone collagen and the lacuna increased around the new trabecula, in which bone cells were clearly observed. Between weeks 8 and 12, the tantalum surface and pores were fully covered with new bone tissue. New trabecula had matured and contacted the material directly, presenting an interwoven, parallel arrangement, which covered the surface and filled the pores. The tantalum and bone formed a solid tantalum-bone direct bond, with no soft tissue layer at the interface (Fig. 4).

**Figure 5:**
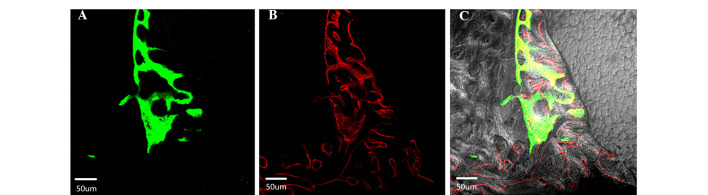
Osteogenesis assessment via laser scanning confocal microscopy revealed a (A) 488-nm calcein fluorescence band and (B) 543-nm alizarin red fluorescence band. (C) Overlaid image of A and B.

Observation by laser scanning confocal microscopy at week 10 after the porous tantalum implantation revealed new bone tissue, which was visible by green fluorescence (calcein) and red fluorescence (alizarin red) at the tantalum-host bone interface and within the pores. The red fluorescence band surrounded the green band. The green and red fluorescent bands were discontinuous in the early stage, but integrated by the late stage (Fig. 5).

## Discussion

The porous tantalum used in the present study was developed by Chongqing Runze Medical Devices Co. Ltd. The appearance of the material is gray, bright and clean. Under SEM, the material exhibits a three-dimensional spatial structure. Under ×500 magnification, pores with a diameter of 200–400 μm are visible on the material surface, which are evenly distributed and interconnected. The trabecula between the pores is rough, presenting a micropore structure with a diameter of 100 μm. The micropore structure is clearly visible under ×1,000 magnification, with a diameter ranging between 50 and 200 μm. Since porous tantalum has a three-dimensional geometric scaffold structure and interconnected honeycomb pores, it offers a large internal surface area, a three-dimensional structure and a good material-cell interface on which osteoblasts are able to adhere, grow and develop (16–18).

The roughness of the porous tantalum increases the cell contact area, improving the adhesive strength of the osteoblasts on the material surface. Tantalum surfaces ground by grit and etched by acid have been shown to promote osteogenesis (19,20). A study by Sagomonyants *et al* (21) indicated that tantalum has useful physical, chemical, mechanical and biological properties, including a fully interconnected porous structure. This nanopore structure has a large impact on osteoblast proliferation, and its rough surface may absorb more large molecules, promoting cell adhesion, proliferation and osteogenic capability. Tantalum also facilitates the expression of osteoblast ALP and collagen type I. In addition, bone tissue ingrowth and pore size are closely associated. The microcolumn structure on the tantalum surface promotes the overall osteogenic response. The high density per volume of the column with a small diameter affords stronger mechanical properties, resulting in more complete ingrowth of bone tissue and better integration with the host tissue following implantation. Thus, tantalum is particularly suitable for clinical application as it cannot be degraded and supports bone function for a long time (8).

In the present study, osteoblasts cultured *in vitro* were seeded onto porous tantalum samples. Observation by SEM showed that the cells adhered to the tantalum surface and pore walls by day 3 of the culture, presenting various morphologies. The cells proliferated further and numerous cells had merged into flakes by day 7, while a number of cells secreted matrix, covering the surface of the material. By day 10, the cells covering the surface had secreted large amounts of matrix, which almost completely covered the material. This result demonstrated that the three-dimensional porous structure of the tantalum material provided not only an ideal space for adhesion and proliferation, but also promoted secretion and infiltration of nutrients and metabolites, which demonstrates that Chinese porous tantalum offers good biocompatibility.

The interaction between osteoblasts and implants is an important focus of research into the compatibility of bone graft materials. The basis of this interaction is the adhesion of cells to the implant material. Cells must interact with the surface of the scaffold, including adhering, extending, migrating, differentiating and proliferating. Adhesion to a material, including metal, can affect the proliferation and differentiation of cells. The interaction between osteoblasts and the metallic material depends largely on the surface properties of the material, which include the geometric structure, porosity and chemistry. The Chinese tantalum implantation material has a three-dimensional structure and interconnected pores of sufficient size, which is beneficial for bone cell adhesion and migration, and thus, appropriate for osteogenesis. Therefore, this material may be used as a bone graft substitute material.

The cytotoxicity test is one of the most important detection indexes prior to the clinical application of biological materials or medical devices, and is also the first test in biological safety evaluation. Currently, there are two methods for detecting cytotoxicity. The first is morphological observation, which involves observing morphological changes and growth to determine whether the material is toxic. The second method is to evaluate viability with an MTT assay, which involves calculating the relative cell proliferation rate based on light absorption indirectly, in order to determine the level of cytotoxicity. Higher cell proliferation rate was associated with lower toxicity of the tantulum material (22). In the present study, detection based on cytotoxicity test standards outside the body from an international biological evaluation system of medical devices showed that osteoblast proliferation increased slowly in the early stage, increased rapidly in the middle stage (days 4–7) and then increased slowly again. In the experimental and control groups, osteoblasts maintained good morphology, with no statistically significant difference observed in the OD values between the groups (P>0.05). Therefore, the results indicated that tantalum ions are not toxic to osteoblast cells. This result was consistent with international standards; thus, Chinese porous tantalum may be safe to implant in animals.

Requirements for bone graft materials include excellent mechanical properties, a porous structure and the capacity to support ingrowth of the host tissue. Compared with other metallic materials, porous tantalum is stable and has a higher porosity, larger pore size and a low elastic modulus (7). Therefore, it is used in hip and knee joint reconstruction surgery and in the production of prostheses (23). In the present study, hard tissue slicing technology combined with specific pathological staining was applied. Light microscopy was used to observe osteogenesis on the surface of the material at the tantalum-bone interface. The results showed that the porous tantalum-bone interface was the most active area of osteogenesis during the early stages of culture. The morphological characteristics of the new bone indicated that the osteoblasts were able to adhere, grow and proliferate in the pores of the material following the implantation of porous tantalum in the host bone, and was accompanied by the ingrowth of small blood vessels. Bone lacunae were observed in the new trabecula, in which new bone cells were clearly visible. By week 12 after implantation, the surface and the pores of the porous tantalum rods were fully covered with new bone tissue, presenting an interwoven arrangement. Similarly to common paraffin slices, hard tissue slices reflect only the local osteogenic state at a particular point and are unable reveal the complete osteogenic process. In the present study, rabbit hips were injected intramuscularly with fluorescein calcein and alizarin red on days 5 and 19 after implantation, respectively. New bone tissue at the tantalum-bone interface and in the pores was observed in the hard tissue slices using laser scanning confocal microscopy at laser wavelengths of 488 nm (calcein) and 543 nm (alizarin red); thus, the osteogenesis process was observed dynamically. On day 5, the green fluorescent band of the immature bone cells marked by calcein was observed, and a red fluorescent band of new bone tissue marked by alizarin red was observed on day 19. The bands were in direct contact with the surface of the tantalum and grew into the pores. These results showed that osteogenesis occurred on the surface of the host bone in the early stage after implantation and that bone formation was time-dependent, which suggests that new bone tissue at the tantalum-host bone interface matures over time, with calcium deposition occurring later. The new bone and host bone integrate and become difficult to distinguish. This form of osteogenesis may explain the existence of a solid combination of porous tantalum and bone, as well as bone conduction to a certain extent.

When the pore diameter of a porous material is 15–40 μm, fibrous tissue has been reported to grow into the interior of the material (24,25). When the diameter reaches 40–100 μm, ingrowth of non-calcified bone-like tissue occurs. When the diameter exceeds 150 μm, the pore structure of the scaffold may fill completely with bone tissue, potentially facilitating an improved osseointegrative effect. Chinese porous tantalum has a high porosity (65–80%) and is able to induce the development of fibrous tissue with rich blood vessels and bone tissue growth inside its pores. Implanted tantalum and host bone may produce a stable connection and integration. These results also further confirm that porous tantalum offers good tissue compatibility and promotes the adhesion, proliferation and differentiation of osteoblasts. Therefore, Chinese porous tantalum is a suitable bone graft substitute material for the construction of three-dimensional structures for bone defect repair. In the present study, porous tantalum was only observed in the animals for three months; therefore, a longer observation period may be tested in future studies. Furthermore, various metal implant materials were not compared in the present study, such as porous titanium material, which may be studied in future research.

## Figures and Tables

**Table I:** Material physical and chemical performance indicators.

Indicators	Range
Density (g/cm^3^)	3.5–7
Porosity (%)	65–80
Pore diameter (μm)	400–600
Elasticity modulus (GPa)	2.0–4.6
Ultimate strength (MPa)	110–210
Yield strength (MPa)	75–120
Compressive strength (MPa)	100–170
Tensile strength (MPa)	80–120
Bending strength (MPa)	80–150
